# Apelin Controls Angiogenesis-Dependent Glioblastoma Growth

**DOI:** 10.3390/ijms21114179

**Published:** 2020-06-11

**Authors:** Anne Frisch, Stefanie Kälin, Raymond Monk, Josefine Radke, Frank L. Heppner, Roland E. Kälin

**Affiliations:** 1 Department of Neuropathology, Charité–Universitätsmedizin, 10117 Berlin, Germany; anne.frisch@charite.de (A.F.); kaelin.stefanie@gmail.com (S.K.); bashfull30@gmail.com (R.M.); Josefine.Radke@charite.de (J.R.); frank.heppner@charite.de (F.L.H.); 2 German Cancer Consortium (DKTK), Partner Site Charité Berlin AND Berlin Institute of Health (BIH), 10178 Berlin, Germany

**Keywords:** Apelin-13, APLN, APLNR, glioblastoma, GBM angiogenesis

## Abstract

Glioblastoma (GBM) present with an abundant and aberrant tumor neo-vasculature. While rapid growth of solid tumors depends on the initiation of tumor angiogenesis, GBM also progress by infiltrative growth and vascular co-option. The angiogenic factor apelin (*APLN*) and its receptor (*APLNR*) are upregulated in GBM patient samples as compared to normal brain tissue. Here, we studied the role of apelin/APLNR signaling in GBM angiogenesis and growth. By functional analysis of apelin in orthotopic GBM mouse models, we found that apelin/APLNR signaling is required for in vivo tumor angiogenesis. Knockdown of tumor cell-derived *APLN* massively reduced the tumor vasculature. Additional loss of the apelin signal in endothelial tip cells using the *APLN*-knockout (KO) mouse led to a further reduction of GBM angiogenesis. Direct infusion of the bioactive peptide apelin-13 rescued the vascular loss-of-function phenotype specifically. In addition, *APLN* depletion massively reduced angiogenesis-dependent tumor growth. Consequently, survival of GBM-bearing mice was significantly increased when *APLN* expression was missing in the brain tumor microenvironment. Thus, we suggest that targeting vascular apelin may serve as an alternative strategy for anti-angiogenesis in GBM.

## 1. Introduction

Glioblastoma (GBM) is the most frequent and most aggressive primary brain tumor [1], and, -despite intensive efforts including recent advances of anti-GBM treatment modalities, there are still only limited therapeutic options, offering patients only an average survival time of one year after diagnosis, at best [2,3]. Hallmarks of GBM biology include strong invasiveness of tumor cells and the abundance of an aberrant vasculature [4]. GBM cells infiltrate normal brain tissue and, as a result, surgical resection is always incomplete [3,5,6]. Regardless of aggressive surgery and radio and chemotherapy, GBM remain uniformly fatal in patients. Hence, alternative therapeutic approaches for treatment against GBM are urgently needed.

The finding that highly vascularized tumors are rapidly growing while tumors with low vessel density are rather dormant led to the idea that tumor angiogenesis was required for tumor progression [7]. Vascular endothelial growth factor—A (VEGFA), which was first identified as a vascular permeability factor—turned out to be a potent endothelial mitogen [8]. The treatment of xenografted mice with a specific monoclonal VEGFA antibody demonstrated that VEGFA was a strong in vivo pro-angiogenic factor and that its blockade suppressed tumor growth [9]. This led to the first successful anti-angiogenic therapy with a humanized monoclonal antibody that blocks VEGFA (bevacizumab) in colorectal cancer and resulted in FDA approval [10]. However, recent clinical studies performed in patients with GBM using bevacizumab (AVAglio, RTOG-0825) did not show an improved overall survival [11,12]. Possible explanations for treatment resistance to bevacizumab are the ability of GBM cells for vascular co-option to obtain access to blood supply [13] or the upregulation of alternative angiogenic factors [14]. In the search for such additional factors involved in tumor angiogenesis, Masiero and colleagues [15] analyzed the expression profile of more than 1000 well-vascularized primary human cancers (head and neck squamous cell carcinomas, breast cancers, and clear cell renal cell carcinomas) for genes correlating with that of several well-recognized angiogenesis signature genes. The angiogenesis core signature they identified contained a set of 40 genes, including the apelin receptor (*APLNR*; also known as APJ). The human *APLNR* gene was coincidently cloned with primers designed to obtain vasopressin receptors in 1993 and found to encode a 7 transmembrane receptor related to the angiotensin II receptor type (*AGTRL1*) [16]. By screening various tissue extracts for ligands of orphan G-protein coupled receptors (GPCR) in an extracellular acidification assay, Tatemoto et al. (1998) [17] identified the apelin isoforms apelin-36, apelin-17, apelin-13, and the pyro-glutamylated (Pyr)apelin-13 as bioactive peptides encoded by the apelin gene (*APLN*). It is now established that the different apelin isoforms are the cognate ligands for APLNR with different binding properties [18].

In our previous work, we described a proangiogenic role of apelin acting as chemoattractant during vertebrate development [19]. That apelin signaling is instrumental for cell attraction was first shown for *APLNR*-transfected cell lines [20,21] and then verified for immortalized and primary vascular endothelial cells [19,22,23], vascular smooth muscle cells [24,25], lymphatic endothelial cells [26], and one tumor cell line (lung adenocarcinoma cell line A549) [27]. The functional importance of apelin in angiogenesis and its epistatic relationship with VEGFA signaling was established in a series of loss- and gain-of-function experiments in *Xenopus* tadpoles [19,22] and further confirmed in other organisms, like zebrafish and mouse [28]. In early vessel development, apelin appears to act in a paracrine fashion to first induce intersomitic blood vessel outgrowth shaping the primitive vascular plexus [19,29,30]. Next, during vessel sprouting, *APLN*-expression gets confined to endothelial tip cells, while the receptor is also found in the stalk cells controlling vessel-guidance and -maturation [19,28,31]. *APLN* can thus be used as a tip cell marker for ongoing angiogenesis and has a role in sprouting angiogenesis in pathology [32]. While *APLNR* mutant mice are born at sub-mendelian ratios, *APLN* knockout (KO) mice are viable and fertile [33,34,35]. The lethality observed in *APLNR*-KO mice is due to growth retardations and cardiac malformations during embryogenesis [36].

By in situ hybridization on GBM patient specimens, we found the first indication of a role for apelin signaling in tumor development. While *APLN* RNA was undetectable and only low level of *APLNR* RNA was found in normal brain vessels, we detected a dramatic upregulation of both within GBM-associated microvascular proliferations, particularly in areas of vessel sprouting and branching as assessed by in situ hybridization [19]. Co-expression of ligand and receptor in the tumor vasculature suggested an autocrine mode of signaling, similar to the one observed during embryonic development [19]. In addition, we also found abundant *APLN* expression in the pseudo-palisading areas of GBM. In these hypoxic regions, *APLN* is co-expressed with VEGFA [19,37], suggesting a cooperative function of apelin and VEGFA in paracrine signaling from tumor to endothelial cells during GBM angiogenesis.

All these data hint towards a vascular function for apelin/APLNR signaling during GBM growth. To study the role of apelin in establishing the structure and function of the GBM vascular beds and its implications to tumor growth, we here used established GBM mouse models. Intracerebral implantation of GBM cell lines with different *APLN* expression levels showed that tumor-derived apelin is required for the formation of the GBM neo-vasculature. Moreover, loss-of-*APLN* in the tumor microenvironment of *APLN*-KO mice showed that *APLN* expression controls patterning of the glioma vasculature. We also show, for the first time, that this sprouting angiogenesis effect is specific to *APLN* gene function as the vascular patterning phenotype was rescued by adding back the bioactive apelin-13 peptide. Furthermore, we found that the loss-of-*APLN* expression in the tumor microenvironment reduced angiogenesis-dependent tumor growth and increased the survival of GBM-bearing mice. Together, our data demonstrates that the endothelial *APLN* signal can serve as an alternative target to reduce GBM growth by specifically blocking sprouting angiogenesis.

## 2. Results

### 2.1. APLN Is Expressed at Variable Levels in GBM Cells and Upregulated in the Pathologic Neo-Vasculature

**Figure 1:**
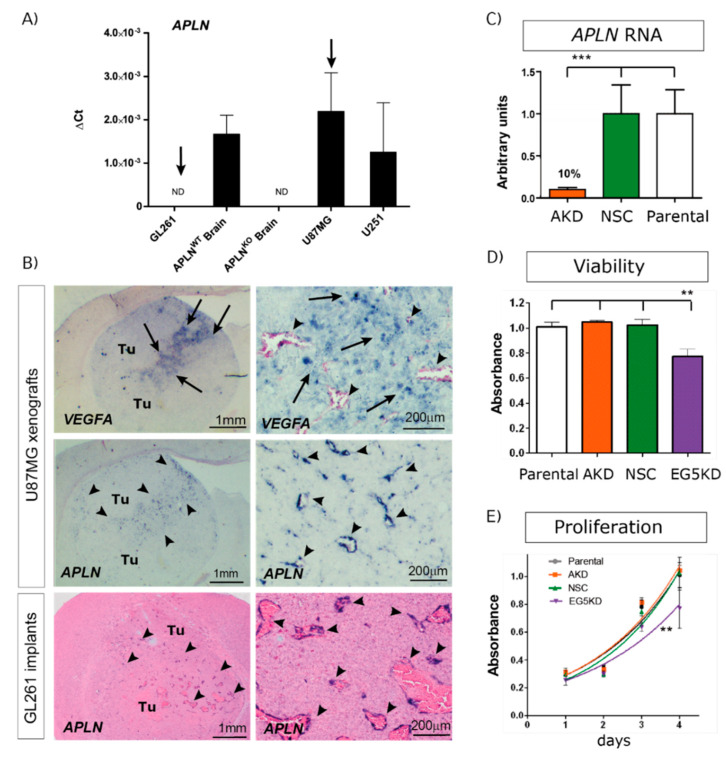
Angiogenic factor apelin (*APLN*) expression in glioblastoma (GBM) cells and the tumor neo-vasculature. (**A**) Expression analysis of *APLN* RNA was performed by qPCR on the human cells U87MG and U251, the murine GL261 cells and on *APLN*^WT^ or *APLN*^KO^ mouse brain tissue. *APLN* RNA expression was not detectable (n.d.) in GL261 cells but high in U87MG cells. (**B**) In situ hybridization against mouse *APLN* showed no expression in implanted mouse GL261 tumor cells but an upregulation of *APLN* in the tumor vessels of GL261 or U87MG gliomas (arrowheads). The right panel is the magnifications within the tumor that is depicted in the overview panel on the left. Note that vascular *APLN* RNA expression is highest in hypoxic regions marked by human vascular endothelial growth factor (*VEGFA*) RNA expression (arrows) in the U87MG xenografts. (**C**) Lentiviral transduction of U87MG parental cells caused shRNAmir-mediated stable *APLN* knock-down (AKD) by 90% (as analyzed by qPCR) compared to the non-silencing shRNA control (NSC) transduced cells. (**D**) Cell viability, as well as in vitro proliferation (**E**), was unchanged in U87^AKD^ cells compared to U87^NSC^ control or untransduced U87MG cells. In contrast, U87 cells transduced with the shRNA against the kinesin EG5 significantly reduced viability and proliferation of U87^E5KD^ cells compared to U87^NSC^ control. Data are obtained from more than 3 independent experiments each and reported as mean +/-SEM; statistical significance (one-way ANOVA plus Bonferroni’s post hoc test) is indicated ** *p* < 0.005, *** *p* < 0.0005. WT = wildtype; KO = knockout.

To study if tumor cell-derived *APLN* controls angiogenesis-dependent GBM growth, we first tested widely used GBM cell lines for *APLN* expression, revealing differing levels of expression. Using *APLN* expression levels in mouse wildtype (WT) brains as a comparison, we characterized the human cell-line U87MG as having high levels of *APLN* expression and the human U251 (formerly known as U373MG) cell line expression as being lower, whilst in the murine GL261 glioma cell-line *APLN* expression was not detectable (n.d.; Figure 1A).

In the next step, we orthotopically implanted human, as well as murine, GBM cells into immunodeficient or immunocompetent mice, respectively, to assess *APLN* expression in in situ pathology. In a previous study, we had found that *APLN* was not only expressed in GBM cells but was also upregulated in the vascular proliferates of the disease [19]. Thus, we also tested our experimental GBM models for vascular *APLN* expression and found that *APLN* expression was upregulated in newly forming tumor vessels (Figure 1B; arrowheads) of the xenografts. Interestingly, the vascular *APLN* RNA signal was highest in the tumor regions where human *VEGFA* RNA was also detected. *VEGFA* expression is a marker for hypoxic tumor areas in pseudopalisading necrosis in GBM. In addition, in murine GL261 glioma, *APLN* expression became localized to newly forming tumor vessels but remained absent from GL261 tumor cells (Figure 1B). To also address the role of GBM cell-derived *APLN*-levels, we selected the high *APLN*-expressing U87MG cell line, in addition, and lentivirally transduced it with shRNA against *APLN* RNA to create tumor cells with stable reduction of *APLN* RNA expression by 90% (U87^AKD^) compared to the non-silencing control (NSC) shRNA-transduced cells (U87^NSC^) or to untransduced parental U87MG cells (Figure 1C). Viability, as well as in vitro proliferation, of the manipulated cells was tested and turned out to be unchanged compared to their parental controls (Figure 1D,E). In contrast, cells transduced with the shRNA against EG5 kinesin (encoded by the Kinesin Family Member 11 gene *KIF11* involved in nuclear division and cell growth [38]) caused reduced viability and proliferation rates (Figure 1D,E).

### 2.2. APLN Expression Is Required for the Formation of the GBM Neo-Vasculature

**Figure 2:**
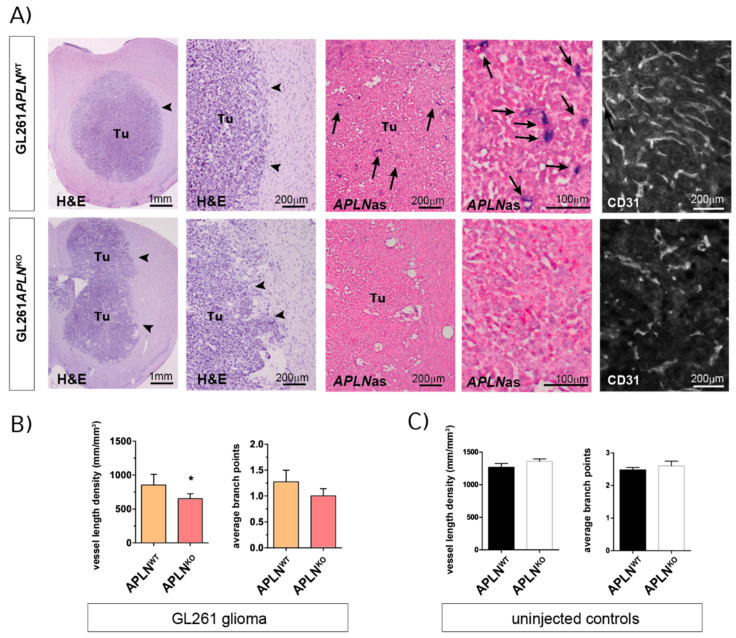
Endothelial *APLN* expression controls formation of a complex GBM vasculature. Orthotopic GL261 implants in *APLN*^WT^ or *APLN*^KO^ mice 21 days post-implantation (dpi). (**A**) The panels on the left indicate the tumor (tumor boarder highlighted by arrowheads) in the right brain hemisphere in overview and close up view on Hematoxylin & Eosin (H&E) sections. The in situ hybridization panels in the middle show the loss-of vascular *APLN* expression (arrows) in the tumor in overview and close up view when comparing *APLN*^WT^ to *APLN*^KO^ mice. The CD31-immunostaining shown in the panel on the right illustrates the reduced vessel density in *APLN*^KO^ mice. (**B**) Vessel length density (VLD) was assessed on CD31 immunofluorescent brain slides and demonstrated a significant decrease in *APLN*^KO^ mice. In addition, vascular complexity measured by the average branch points (ABP) was reduced in *APLN*^KO^ as compared to *APLN*^WT^. (**C**) In the healthy brain, VLD and ABP do not differ in *APLN*^KO^ compared to *APLN*^WT^ mice. Data of *n* = 9 *APLN*^WT^ vs. 6 *APLN*^KO^ mice are reported as mean +/-SEM; statistical significance (students test) is indicated * *p* < 0.05.

In agreement with a range of previous reports [39,40], we observed that orthotopic implantation of GL261 cells generated compact glioma mass 21 days post-implantation (dpi) with an abundant but aberrant tumor vasculature (Figure 2A, upper panel). To investigate the impact of *APLN* expression derived from the tumor microenvironment, namely *APLN* expressed on endothelial tip cells [19,28,31,32] during sprouting angiogenesis (Figure 1B), we implanted GL261 cells into *APLN*^KO^ mice. We found that the tumor vasculature was significantly attenuated compared to *APLN*^WT^ control mice (Figure 2A,B). Specifically, we found by stereological assessment of the tumor tissues that the vessel length density (VLD), as a read-out for the extent of tumor vascularization independent of tumor-size [41,42,43], was 856 mm/mm^3^ in controls, while this number significantly decreased to 653 mm/mm^3^ in *APLN*^KO^ glioma (Figure 2B). Note that in situ hybridization for mouse *APLN* RNA shows that *APLN* is absent in tumor cells, as well as in the GBM microenvironment, of *APLN*^KO^ mice, while *APLN* expression in *APLN*^WT^ mice is specifically upregulated in endothelia of the tumor neo-vasculature (Figure 2A; arrows). Interestingly, the VLD determined in GL261 implants is smaller than the values observed for a healthy tumor-free brain. In the caudate putamen, which is the brain region targeted by the tumor cell implantation, we measured a VLD of 1269 mm/mm^3^ for *APLN*^WT^ mice and 1359 mm/mm^3^ for *APLN*^KO^ mice, without the difference being significant (Figure 2C). Similar values for VLD were also previously reported for the tumor-free hippocampus (1100 mm/mm^3^) of adult mice [44]. This finding of reduced vessel density in GBM as compared to healthy brain can be explained by the neoplastic growth and the higher cell density inside tumors. While the VLD of tumor-free brain is unchanged in *APLN*^KO^ mice, the VLD of the glioma is reduced in *APLN*^KO^ mice compared to *APLN*^WT^ controls. In addition, loss-of host-derived *APLN* also resulted in reduced vascular complexity, serving as an indicator of the extent of angiogenic sprouting [45]. We found that the average number of vessel branch-points (ABP), which measured 1.25 in *APLN*^WT^ glioma, was reduced to 1 in tumor vessels of *APLN*^KO^ mice. In addition, the ABP values in the tumor were thus lower than the values observed in tumor-free striatum (Figure 2C; ABP = 2.5 in *APLN*^WT^ and 2.6 in *APLN*^KO^). This means that the vascular complexity in *APLN*^KO^ GBM is low to nearly linear, and this indicates that no more angiogenic sprouting took place during tumor growth.

Together, these results show that autocrine signaling of endothelial apelin to *APLNR*-expressing vessels is required for sprouting angiogenesis to take place and shape the complex tumor neo-vasculature.

### 2.3. Paracrine and Autocrine APLN Signaling Controls GBM Angiogenesis

**Figure 3:**
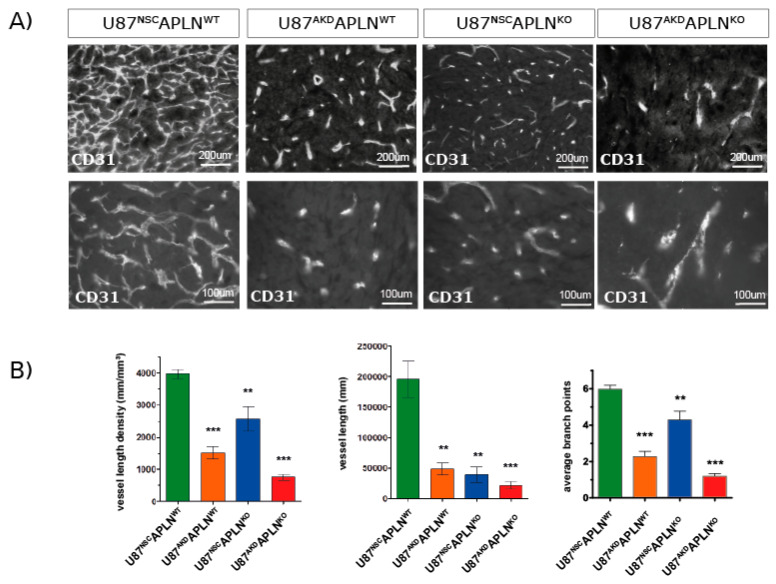
GBM-and endothelial cell-derived *APLN* are both controlling sprouting angiogenesis. (**A**) U87MG was implanted into immunodeficient mice and grown to big xenografts within 28 dpi. Fluorescent immunostaining for CD31 was performed and is depicted in an overview and a close up view for every xenograft. (**B**) The microvasculature in the green fluorescent protein (GFP)-positive tumor area was analyzed by stereomorphology. In comparison to U87^NSC^*APLN*^WT^ controls (*n* = 8), VLD in U87^AKD^*APLN*^WT^ xenografts (*n* = 13) was reduced by 62% and vessel length by 75%. Additional loss-of-*APLN* expression in the tumor neo-vasculature of the *APLN*^KO^ mouse (U87^AKD^*APLN*^KO^, *n* = 10) lead to further reduction of the tumor vasculature. VLD was also reduced when only the tumor cells express *APLN* (U87^NSC^*APLN*^KO^, *n* = 9) but was the highest of all manipulated xenografts. While vascular complexity measured by vascular branch points (ABP values are 6, 2.3; 4.3; and 1.2, respectively) reflects the results seen by VLD, the total vessel length was highly reduced in all three xenografts with reduction of *APLN* expression. Data are reported as mean +/−SEM; statistical significance (one-way ANOVA plus Bonferroni´s post hoc test) is indicated ** *p* < 0.005, *** *p* < 0.0005.

Next, we wanted to study the contribution of GBM cell-derived *APLN* to tumor angiogenesis and thus we compared vascular patterns generated by U87MG cells with a stable knock-down for *APLN* (U87^AKD^) to non-silencing control cells (U87^NSC^; Figure 1C–E). The modulation of human GBM-derived *APLN* is pathologically meaningful in mouse models as the amino acid sequence of the bioactive peptide apelin-13 of human or mouse origin is 100% identical [19]. Four weeks after tumor-induction with U87^NSC^ cells in immune-deficient WT mice (U87^NSC^*APLN*^WT^), we observed an extremely dense tumor vasculature (Figure 3A) with a VLD of 3937 mm/mm^3^ (Figure 3B), while the VLD of U87^AKD^-induced GBM in WT mice (U87^AKD^*APLN*^WT^) was 1519 mm/mm^3^ (62% reduced as compared to U87^NSC^*APLN*^WT^). Injection of U87^NSC^ into *APLN*^KO^ mice (U87^NSC^*APLN*^KO^) resulted in a VLD of 2601 mm/mm^3^, whereas combined ablation of *APLN* expression in U87MG cells and in the host (implantation of U87^AKD^ cells into immune-deficient *APLN*^KO^ mice; U87^AKD^*APLN*^KO^) largely blocked tumor angiogenesis and resulted in a VLD of 766 mm/mm^3^, which is lower than in tumor-free brain-tissue (Figure 2C and Figure 3B). While vessel branching reflects the picture observed by VLD assessment, the total vessel length (that is the VLD multiplied by the individual tumor volume) was highly reduced in all three xenografts with reduction of *APLN* expression (Figure 3B). At the same time, the change in *APLN*-levels did not affect the extent of pericyte cell coverage as assessed by CD31/Desmin double-immunofluorescence (Figure S1A). BrdU in vivo labeling of the tumor vasculature further demonstrated that endothelial cells are still able to proliferate upon *APLN* level reduction (Figure S1B).

These data showed, on one hand, that the high expression of *APLN* in the U87MG (and U87^NSC^ control) cells induces a very dense and complex tumor vasculature as compared to U87^AKD^ cells depleted in *APLN*-expression, as well as the APLN-non expressing GL261 cells. On the other hand, the loss-of-*APLN* in both GBM cell models in the tumor microenvironment decreased vascular sprouting to a level even lower than the vessel density observed throughout the healthy brain.

Together, these experiments indicate that paracrine (from the tumor) and autocrine (from the host microenvironment) *APLN* release has profound effects on the extent of GBM angiogenesis and thus the vascular pattern generated.

### 2.4. Apelin-13 Specifically Controls Vessel Density in the GBM Neo-Vasculature

**Figure 4:**
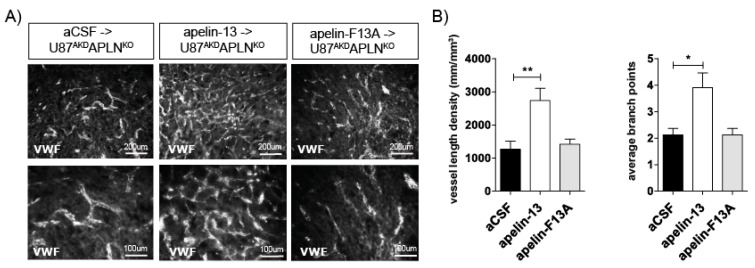
Apelin peptide rescues the vascular loss-of-function phenotype. (**A**) Intracerebral infusion of 30 µg of apelin-13 peptide (*n* = 9) increased glioma angiogenesis in U87^AKD^*APLN*^KO^ xenografts compared to infusion of artificial cerebrospinal fluid (aCSF, *n* = 8) or apelin-F13A (*n* = 6) antagonist as shown by von Willebrand factor (VWF) staining. (**B**) Quantification on a Stereoinvestigator resulted in a VLD of 1269 mm/mm^3^ in U87^AKD^*APLN*^KO^ xenografts infused with aCSF only; 2573 mm/mm^3^ with the *APLN* receptor (*APLNR*) agonist apelin-13 peptide or 1419 mm/mm^3^ with the *APLNR* antagonist apelin-F13A. ABP obtained were 2.2; 3.9 and 2.2, respectively. Data are reported as mean +/−SEM; statistical significance (one-way ANOVA plus Bonferroni´s post hoc test) is indicated. * *p* < 0.05, ** *p* < 0.005.

In the following experiment, we addressed the question if *APLN*-deficiency in our mouse model (i.e., in the *APLN*^KO^ mice) would lead to, e.g., developmentally regulated alterations in the signal transduction pathways controlling angiogenesis or if *APLN*-responsiveness by *APLN*R is maintained in the absence of the ligand. Therefore, we infused the bioactive apelin-13 peptide into the U87^AKD^*APLN*^KO^ xenograft model and were indeed able to obtain a partial rescue of the tumor neo-vasculature increasing VLD from 766 mm/mm^3^ (infusion of artificial cerebrospinal fluid; aCSF control) to 2573 mm/mm^3^ (Figure 4). In contrast, the C-terminally mutated peptide apelin-F13A (which had been used as an antagonist for physiological functions on *APLNR* before [46]) was not able to rescue tumor angiogenesis. Instead, when apelin-F13A was infused into U87^NSC^APLN^WT^ GBMs expressing endogenous APLN levels, it significantly reduced VLD working as a competitive antagonist on APLNR (Figure S1C). Together, this part of our study supports the notion that *APLN*^KO^ mice are devoid of endogenous *APLN* but can respond to exogenous or tumor-derived *APLN*. This is a prerequisite to explain the gradual anti-angiogenic responses to *APLN*-ablation in our GBM models. Here, we observed that *APLN* -deficiency in the GBM-cells or in the host generates a tumor mass with a reduced vascularization, as compared to tumor with unmodulated *APLN* levels. Ablation of *APLN* in both the GBM cells and in the tumor-bearing animals resulted in a striking cooperative anti-angiogenic effect. The partial rescue of the vascular GBM phenotype by addition of apelin-13 peptide to *APLN*-depleted xenografts shows that these effects are specifically mediated by apelin-signaling.

### 2.5. Loss of APLN Reduces Angiogenesis-Dependent Tumor Growth

**Figure 5:**
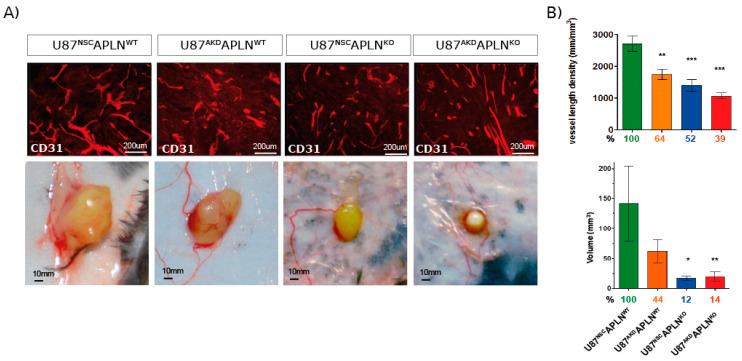
Apelin is required for compact tumor growth. Mice were inoculated subcutaneously with U87 cells and grown for 28 dpi. (**A**) Tumor volumes were measured, and vessel density was quantified on CD31-immunostained sections. Representative pictures of subcutaneous tumors are shown. (**B**) VLD and tumor volume was significantly attenuated upon reduction of *APLN* expression as compared to U87^NSC^*APLN*^WT^ controls (percentages compared to the control group are indicated). Tumor volumes were reduced to 50% in *APLN* knockdown U87^AKD^*APLN*^WT^ xenografts. Further reduction to less than 15% was observed in *APLN*^KO^ mice. Number of cell implantations for U87^NSC^*APLN*^WT^, U87^AKD^*APLN*^WT^, U87^NSC^*APLN*^KO^, U87^AKD^*APLN*^KO^ were *n* = 38, 31, 18, 24, while tumor take was 87%, 90% 72%, 50%, respectively. For VLD analysis *n* = 7, 6, 7, 6 of the respective xenografts were analyzed. Data are reported as mean +/−SEM; statistical significance (one-way ANOVA plus Bonferroni’s post hoc test) is indicated * *p* < 0.05, ** *p* < 0.005, *** *p* < 0.0005.

To compare the effect of *APLN* on tumor growth, we performed a classical test for in vivo anti-angiogenic pharmacological function [9,47]. By generating subcutaneous xenografts, we found that *APLN* knockdown in tumor cells, as well as the loss-of-*APLN*, in the tumor microenvironment significantly reduced VLD by 36% (U87^AKD^*APLN*^WT^) and 48% (in U87^NSC^*APLN*^KO^), respectively, as compared to U87^NSC^*APLN*^WT^ controls (Figure 5). The depletion of both, tumor and host *APLN* in U87^AKD^*APLN*^KO^ xenografts further reduced VLD by 61% as compared to U87^NSC^*APLN*^WT^ control. Interestingly, the tumor volume was reduced by 50% when tumor-cell derived *APLN* was depleted compared to *APLN*-expressing control cells. Strikingly, if *APLN* was missing in the tumor microenvironment, the tumor volume decreased to less than 15% compared to WT controls. We also assessed if the tumor cells were growing equally well in all mice. We found that tumor take was similar for *APLN*-knockdown (87% for U87^AKD^*APLN*^WT^) compared to U87^NSC^ control cells (90% for U87^NSC^*APLN*^WT^) when implanted in WT mice and only slightly reduced when *APLN* was missing in the tumor microenvironment in *APLN*^KO^ but still present in the tumor cells (72% for U87^NSC^*APLN*^KO^ xenografts). However, when *APLN* expression was fully depleted as in U87^AKD^*APLN*^KO^ xenografts, only 50% of tumor cell implantation led to successful tumor growth.

This experiment demonstrated that apelin is important for tumor angiogenesis and that compact subcutaneous tumors depend on the apelin-induced neo-vasculature for their growth.

### 2.6. Loss-of-APLN Expression in the Tumor Microenvironment Increases Survival of Glioma-Bearing Mice

**Figure 6:**
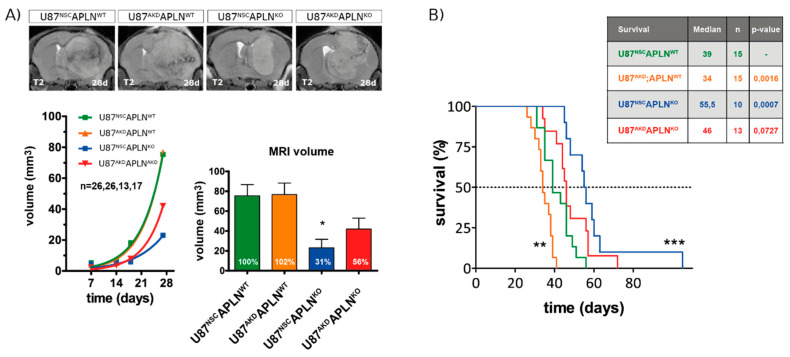
Loss-of-*APLN* in the tumor microenvironment increases survival of GBM mice. Human U87 cells modulated for *APLN* levels were orthotopically implanted into *APLN*^WT^ or *APLN*^KO^ mice. (**A**) T2-weighted magnetic resonance imaging (MRI) was performed weekly and MRI volumes were measured producing an in vivo growth curve for every xenograft. After 28 days, U87^NSC^ or U87^AKD^ cells in *APLN*^KO^ mice showed reduced tumor volume by 69% and 44%, respectively, compared to U87^NSC^*APLN*^WT^ controls. Number of mice per group are indicated. Data are reported as mean +/−SEM; statistical significance (one-way ANOVA plus Bonferroni’s post hoc test) is indicated * *p* < 0.05. (**B**) A mouse survival experiment with orthotopically implanted tumor cells was performed and mice sacrificed at humane endpoints. Median survival differed significantly in U87^AKD^*APLN*^WT^ (shorter survival) and U87^NSC^*APLN*^KO^ (longer survival) mice compared to U87^NSC^*APLN*^WT^ control mice. U87^AKD^ xenografts in *APLN*^KO^ mice showed a trend to longer survival. Number of mice per group are indicated. Survival data are shown as Kaplan-Meier Curves and significant differences between the experimental groups and the U87^NSC^*APLN*^WT^ control group assessed by long-rank (Mantel-Cox) test is given in the table and is indicated in the graph by * *p* < 0.05, ** *p* < 0.01, *** *p* < 0.001.

Finally, to assess the impact of apelin-controlled tumor angiogenesis on survival, we implanted the human GBM cells into *APLN*^WT^ or *APLN*^KO^ mice orthotopically. To follow in vivo tumor growth over time, we performed longitudinal magnetic resonance imaging (MRI) and found that all four groups of xenografts expanded exponentially however at a lower rate in *APLN*^KO^ mice (Figure 6A). At 28 dpi, the U87^NSC^*APLN*^KO^ glioma showed a significantly smaller tumor volume of 31% compared to U87^NSC^*APLN*^WT^ tumors (defined as 100%). These results are pathologically relevant as we could confirm them in the survival experiment in which the *APLN*^KO^ mice bearing U87^NSC^*APLN*^KO^ xenografts survived longest (Figure 6B). Survival of this group increased by 42.3% compared to the U87^NSC^*APLN*^WT^ control group. Surprisingly, *APLN*^WT^ mice receiving the apelin-deficient xenografts (U87^AKD^*APLN*^WT^) survived by 14.7%, significantly shorter than controls (U87^NSC^*APLN*^WT^). Interestingly, when *APLN* levels were decreased stepwise in the tumor microenvironment and the tumor cells, the cell behavior of the GBM cells changed towards increased invasiveness (Figure S2).

In summary, we found that the absence of *APLN* expression from the tumor microenvironment showed a beneficial effect on the survival of GBM-bearing mice.

## 3. Discussion

In this study, we show, for the first time, that apelin peptide specifically and directly controls in vivo GBM angiogenesis. Moreover, we demonstrate that compact GBM growth depends on apelin-directed angiogenesis. Interestingly, the absence of apelin upregulation in the tumor vasculature of *APLN*^KO^ mice led to smaller tumor volume and a significant extension of the survival time for GBM-bearing mice. The results of our study fit with previous in vivo observations made, where ectopic apelin overexpression enhanced angiogenesis and tumor cell proliferation in subcutaneous tumor models [26,48,49]. Moreover, recent results obtained in a mouse model of breast cancer showed that loss-of-*APLN* markedly reduced tumor angiogenesis leading to impaired tumor growth and, consequently, improved survival of these animals [50,51].

In line with these findings, we found in our GBM mouse model that the absence of *APLN* expression from the tumor vasculature showed a beneficial effect on the survival of GBM-bearing mice; however, the reduction of *APLN* expression from the tumor cell had an ambiguous effect on in vivo GBM volume. Specifically, we found that tumor-derived, as well as ectopically infused, apelin-13 could rescue partially the vascular phenotype. However, the tumor volume did not increase in these models (Figure S2D). Thus, vascular *APLN* expression seems to be essential for the formation of a mature tumor neo-vasculature that supports GBM growth. One explanation for this finding might be that the apelin-peptide is present in many different isoforms that could confer additional biological effects [17,52].

Another possibility could be that certain effects might be mediated by the second APLNR ligand APELA that was recently identified to be expressed in GBM [53]. *APELA* expression was associated with poor patient survival and correlated with glioma grade. APELA had been identified as an early APLNR ligand during embryogenesis being essential for early cardiovascular development and controlling cellular movements through APLNR [54,55]. In GBM, *APELA* expression was detected in stem cell niches and may thus drive tumorigenesis by supporting GSC growth [53]. A role in GBM angiogenesis has not been described yet.

Previously, ectopic expression of *APLN* in subcutaneous tumors was described to stabilize tumor vessels and support vascular maturation [56]. Moreover, overexpression of *APLN* reduced the leakiness of vessels in models of ischemia [35]. Our finding that the in vivo volume assessed by MRI was smallest when *APLN* was expressed by the tumor cells, but not by the vessels, might be explained by improved tightness of the neo-vasculature and, consequently, improved function and reduced formation of brain edema. In contrast, the loss-of-*APLN* expression in the tumor cells and the tumor vessels may lead to less functional vessels that finally induce the tumor cells to change their growth behavior. Indications for such a change in tumor cell behavior we recently found in a study of highly invasive glioblastoma stem-like cells [57]. Using these infiltrative GBM mouse models, we found increased in vivo, as well as in vitro, invasion, when the tumor cells lost autocrine apelin signaling [57]. Interestingly, U87^AKD^ cells with low apelin expression also showed increased invasion in vitro as compared to U87^NSC^ cells [57]. Thus, we re-inspected the U87MG xenografts (Figure S2A–C) and found that single invasive cells were detectable detaching from the compact GBM mass when *APLN* expression was knocked down in tumor cells (U87^AKD^). Hence, the notoriously low invasiveness that is generally observed with the U87MG cell line (Figure 1B) seemed to be increased upon loss-of tumor-derived apelin/APLNR signaling. We also observed in our second immunocompetent GBM model, the *APLN*-non-expressing GL261 cells, that GBMs growing in *APLN*^KO^ mice exhibited a drastic increase in glioma cell-invasion (Figure 2A; see arrowheads in Gl261 *APLN*^KO^ samples) as compared to controls. While tumor volume slightly decreased, the invasive score significantly increased from 1.2 to 2.8 (Figure S2E). Moreover, by confocal microscopy, we observed that the GBM cells in the invasive U87^AKD^*APLN*^KO^ GBMs adhere to remaining tumor vessels instead of being equally distributed amongst the highly dense vascular network in the U87^AKD^*APLN*^KO^ control GBMs. And, finally, we found in a qPCR screen for angiogenesis and invasion-related genes that loss-of-apelin directed angiogenesis led to a change in their expression Figure S2F). Marker gene expression levels for angiogenesis like *KDR*, *VEGFA*, *FGF2*, *HIF1a*, and *APLN* itself were decreased. Instead *Timp1*, the inhibitor of the matrix metalloproteinase 2 *MMP2*, was downregulated in U87^AKD^*APLN*^KO^ GBMs compared to the wildtype situation, and *MMP2* itself, a proteinase degrading the extracellular matrix to support tumor cell invasion, was upregulated. Taken together, it seems that *APLN*-deficient GBM cells can change their cellular behavior to adhere to the remaining tumor vessels supporting tumor growth by increased invasiveness into the tumor-free brain. Such a shift towards a more invasive behavior of the tumor might be a reason for GBM recurrence after VEGFA-centered anti-angiogenic therapy, in addition to utilizing alternative pro-angiogenic factors [14], as previously observed in glioma models after blockage of angiogenesis by inhibition of VEGFA signaling [58,59,60]. We also showed that anti-VEGF therapy resulted in decreased *APLN* expression in the glioblastoma-stem-like model, resulting in increased tumor cell invasiveness [57]. To avoid this pro-invasive side effect, we used an alternative approach to target apelin/APLNR signaling by infusion of APLNR antagonists [57]. We, and others, found that the use of such competitive antagonistic peptides, like apelin-F13A or MM54, indeed led to reduced vascularization (Figure S1C), attenuated tumor progression, and increased survival in GBM mouse models, as well as models of breast cancer [51,57,61]. In the breast cancer model, tumor cell invasion was also changed and led to reduced metastatic growth [51]. What hampers the clinical application of such a peptidic agent is their usually low penetration into the target tissue.

Moreover, clinical trials of GBM therapies have highlighted the need to identify predictive markers for clinical outcomes of new therapeutic strategies [2,62]. *APLN* expression levels inversely correlate with overall survival in breast, colorectal, and lung cancer by directly correlating with increased angiogenesis in these tumors [48,51,63,64]. Furthermore, *APLNR* is a marker of increased angiogenesis in many cancer types [15]. However, in GBM, we found that the ratio between *APLN* and *APLNR* expression is an important indicator of therapy success, as low *APLN* and high *APLNR* expression correlated with increased GBM cell invasiveness [57]. Interestingly, targeting vascular APLNR by conditional ablation of APLNR-positive tumor vessels led to a significant reduction of tumor growth [65].

Thus, an overall depletion of apelin in GBM might not be the therapy of choice. Instead, together with our here-presented results, we propose that a more targeted-strategy to specifically deplete vascular apelin (e.g., using an apelin-specific antibody delivered intravenously) can open a new treatment avenue. This strategy would specifically block sprouting angiogenesis by not targeting the established brain vasculature and may lead to additional normalization of the tumor vasculature [50] also in GBM. In addition to the anti-angiogenic effect such an approach would circumvent the increased GBM cell invasion and carry the potential to reduce tumor hypoxia and edema formation, as well [66].

In summary, we found that apelin expression correlated with enhanced vascularization in GBM. Reduction of apelin expression in tumor cells attenuated tumor angiogenesis and ectopic infusion of apelin-13 peptide specifically reversed this vascular GBM phenotype. Moreover, loss-of-*APLN* in the mouse brain further reduced GBM vessel density below normal levels that are observed in the striatum of a healthy mouse brain, underlining the switch to an avascular tumor growth. By this blockade of *APLN*-dependent tumor angiogenesis, tumor growth was reduced and survival of GBM-bearing mice was, in turn, increased. Thus, we believe that targeting vascular apelin/APLNR signaling in the GBM microenvironment specifically offers a promising new opportunity to overcome resistance to anti-angiogenic therapy, as observed by VEGFA blockade, in GBM.

## 4. Materials and Methods

### 4.1. Cell Culture

U87MG and U373 cells were obtained from the American Type Culture Collection (ATCC), GL261 cells were obtained from the National Cancer Institute, NCI-Frederick (Tumor Cell Repository) and all cells were cultured under adherent conditions in DMEM containing 1× MEM non-essential amino acids, 5% penicillin-streptomycin and 10% FBS (all Thermo Fisher Scientific, Waltham, MA, USA). All cell lines were maintained at 37 °C in a humidified atmosphere of 95% O_2_ and 5% CO_2_.

### 4.2. Cell Transduction

For gene silencing of U87MG cells, lentiviral shRNAmir constructs for *APLN* (AKD; cat. RHS4430: V3LHS_401190), *EG5* positive control (EG5KD; cat. RHS4480), or non-silencing control (NSC; cat. RHS4346) were produced in HEK293T cells using the TransLenti Viral GIPZ Packaging System (cat. TPLP4614, all, Dharmacon GE Life Sciences, Lafayette, CO, USA) according to the manufacturer´s instructions. Virus particle-containing supernatant was harvested two days after transfection, filtered with a 0.22 μm filter to avoid cellular contamination and stored at −80 °C. U87MG cells were detached with Trypsin/EDTA (Merck Millipore, Burlington, MA, USA). Eight by ten [4] cells were incubated with 500 μL of virus particles with an multiplicity of infection of 0.6–0.7 for six hours in a 24-well plate; then, 1 mL of medium was added, and cells were left overnight at 37 °C. The day after, cells were centrifuged and resuspended in fresh medium. After cell recovery, selection with puromycin (Sigma Aldrich, St.Louis, MO, USA) was performed for up to three weeks. Concentration of antibiotics had been previously determined by a kill curve. More than 99% efficiency of transduction/selection was confirmed by FACS and immunofluorescence.

### 4.3. Animals

All experiments were performed in compliance with the National Guidelines for Animal Protection, Germany, with approval of the local animal care committee of the “Landesamt für Gesundheit und Soziales” (LaGeSO) in Berlin, and every experiment was conducted after the guidelines of the UK Co-ordinating Committee on Cancer Research [67]. APLN^KO^ mice were obtained from J.P. (Vienna, Austria) [34] and crossed to Rag2^KO^ mice (B6.129S6-Rag2^tm1Fwa^) [68] kindly provided by Prof. G. Willimsky/Charité–Univeritätsmedizin Berlin previously purchased from Taconic (Rensselaer, NY, USA) (all on C57Bl/6J background). All mice were kept in a 12 h light/dark cycle with ad libitum access to food and water. Mice were sacrificed at defined presymptomatic time points or at humane end-point for the survival experiment.

### 4.4. Tumor Implantation

Mice were anesthetized with 7 μL/g of body weight of a mixture of Xylazine (Rompun 2%; Bayer Health Care, Leverkusen, Germany) and Ketamin (Ketavet; Zoetis, Berlin, Germany) in 0.9% NaCl, immobilized on a stereotactic frame (David Kopf Instruments, Tujunga, CA, USA) in flat-skull position and kept warm. A midline incision was made with a scalpel. One by ten [5] cells/μL in medium without supplements were implanted by stereotactic injection 1 mm anterior and 1.5 mm right to the bregma with a 22-gauge Hamilton syringe (Hamilton, Bonaduz, Switzerland) after drilling a whole into the skull with a 23G needle. At a depth of 4 mm, cells were slowly injected within 2 min and, after a settling period of another minute, the needle was removed in 1 mm step/minute. The incision was sutured and patched with Opsite spray dressing (Smith&Nephew, London, UK).

### 4.5. Intracerebral Drug Application

One day before implantation, micro-osmotic pumps were filled with 30 μg pyr-apelin-13 or apelin-F13A for 14 days (micro-osmotic pumps cat. 1002; Alzet; Charles River) in artificial cerebrospinal fluid (aCSF; as described by Alzet) or aCSF alone to be primed overnight in aCSF at 37 °C. Pump implantation was performed under anesthesia using the brain infusion kit 3 (Alzet) inserting the needle into the previously drilled hole after initial orthotopic tumor establishment as observed by magnetic resonance imaging. At the end of pump performance, animals were sacrificed.

### 4.6. Magnetic Resonance Imaging

MRI was performed using a 7 Tesla rodent scanner (Pharmascan 70⁄16AS, Bruker BioSpin Bruker, Billerica, MA, USA) with a 16 cm horizontal bore magnet and a 9 cm (inner diameter) shielded gradient with a H-resonance-frequency of 300 MHz and a maximum gradient strength of 300 mT/m. For imaging, a ^1^H-RF quadrature-volume resonator with an inner diameter of 20 mm was used. Data acquisition and image processing were carried out with the Bruker software Paravision 5.1. During the examinations, mice were placed on a heated circulating water blanket to ensure constant body temperature of 37 °C. Anesthesia was induced with 3% and maintained with 1.5–2.0% isoflurane (Forene, Abbot) delivered in 0.5 L/min of 100% O_2_ via a facemask under constant ventilation monitoring (Small Animal Monitoring & Gating System, SA Instruments, Stony Brook, NY, USA). For imaging the mouse brain, T2-weighted 2D turbo spin-echo sequence was used (imaging parameters for T2 TR/TE = 4200/36 ms, RARE factor 8.4 averages). Twenty axial slices with a slice thickness of 0.5 mm, a field of view of 2.60 × 2.60 cm and a matrix of 256 × 256 were positioned over the brain. Calculation of lesion volume was carried out with the program Analyze 5.0 (AnalyzeDirect, Inc., Mayo Clinic, MI, USA). The hyperintense tumor areas were assigned with a region of interest tool. This enables a threshold-based segmentation by connecting all pixels within a specified threshold range about the selected seed pixel and results in a 3D object map of the whole tumor region. Further, the total volume of the whole object map was automatically calculated.

### 4.7. HE Tumor Volume Analysis

Tumor volumes were obtained from Hematoxylin & Eosin (H&E)-stained tumor sections measuring the area of every 9th section and calculated by the Cavalieri Method [69].

### 4.8. Quantitative PCR

RNA extraction was performed using Trizol (Thermo Fisher Scientific) according to the manufacturer´s instructions. One microgram total RNA was reverse-transcribed into cDNA using QuantiTect Reverse Transcription Kit (Qiagen) and the cDNA was analyzed by quantitative PCR using TaqMan Gene Expression Assays for *APLN* (Hs00936329_m1; Mm00443562_m1; Rn00581093_m1), *EG5* (Hs00189698_m1), and *GAPDH* (Hs99999905_m1; Mm99999915_g1; Rn01775763_g1) with TaqMan Gene Expression Master Mix (Cat. 4369016) in a StepOnePlus Instrument (all Thermo Fisher Scientific). Samples were amplified with the standard running method provided by StepOne Software v2.2.2, increasing the cycle numbers to 45. In every run, gene-of-interest expression levels were normalized to the house-keeping gene GAPDH.

### 4.9. Viability and Proliferation Assays

Six thousand cells/well were plated in 96-well plates in DMEM-F12 medium on day 0. Cell viability was measured after 24, 48, 72, and 96 h using a MTT assay (CellTiter 96 Non-Radioactive Cell Proliferation Assay, Promega, Fitchburg, WI, USA) for cell metabolic activity according to manufacturer´s instruction, incubating the cells one hour with the “Stop Mix” solution. Absorbance was measured with Versa Max microplate reader and SoftMax Pro software (Molecular Devices) with a reference wavelength of 630 nm. Background absorbance from wells containing no cells was subtracted from all measurements, and six replicate samples were used in each experiment. Three experiments per cell type were performed.

### 4.10. In Situ Hybridization

Solutions were prepared with RNAse free water and sterilized. Sections on slides were deparaffinized by serial passages into Roti-Histol and graded alcohol (100–25%). Tissue was permeabilized with 10 min incubation in 10 μg/mL of Proteinase K (PeqLab, VWR). Slides were fixed for 10 min in 4% paraformaldehyde (PFA) and blocked for 10 min with Acetic Anhydride (0.25%; Sigma Aldrich) in Triethanolamine (1.5%; Sigma Aldrich). Sections were dried for 2 h at room temperature, then incubated overnight at 65 °C in a humidified chamber with DIG-labeled (DIG RNA labeling; Roche Diagnostics) antisense or sense probes at a final concentration of 7 μg/mL, and diluted in a hybridization solution containing ssDNA (100 μg/mL, Salmon sperm DNA; Ambion, Thermo Fisher Scientific) to mask unspecific binding and co-precipitant RNA (100 μg/mL, Yeast RNA; Ambion). RNA probes were generated from human VEGFA and mouse *APLN* cDNA as previously described [19]. Probe-containing hybridization solution was boiled at 95 °C for 10 min before application. On day two, unspecific signal was removed with graded stringency washes with saline sodium citrate from 20× to 0.1× and incubated with alkaline phosphatase conjugated anti-DIG antibody (Roche Diagnostics) overnight at 4 °C. On day three, slides were washed in PBT (0.1% Tween in 1×PBS) and incubated with BCIP/NBT substrate (Vector Laboratories, Burlingham, CA, USA) at 37 °C for up to four days. For counterstaining with Eosin, slides underwent serial passages in graded alcohol (70–100%) till Roti-Histol and were then mounted with Entellan (Merck Millipore). Pictures were taken under an Axioskop2 microscope with Axiocam 105 Color and Axiovision SE64 Rel. 4.9 software (Carl Zeiss, Jena, Germany).

### 4.11. Immunofluorescence and Vessel Density Quantification

Mice were transcardially perfused under Narcoren (Merial) anesthesia with 1x PBS followed by 4% phosphate buffered PFA. Brains were post-fixed for two days in 4% PFA and then left in 30% sucrose for at least 24 h at 4 °C. Freezing was performed embedding the tissue in Cryomatrix (Thermo Fisher Scientific) and brains were preserved at −20 °C. Tissue was sectioned horizontally in 40 μm-thick slices on a microtome. Floating sections were blocked for 1 h at room temperature in 1x PBS containing 5% normal donkey serum (NDS; cat. 017-000-121; Jackson Immuno-Research,) and 0.3% Triton-X (cat. 93418; Fluka) and incubated overnight at 4 °C with primary antibodies rat anti-CD31 (1:50, cat. 550274; BD Biosciences) or rabbit anti-VWF (1:400, cat. A0082; Dako, Agilent Technologies, Santa Clara, CA, USA). The next day, sections were incubated for 3 h at room temperature with the secondary antibodies biotin donkey anti-rabbit, anti-rat (1:250, cat. 711-065-152; 712-065-150), and/or 2 h at room temperature with Streptavidin-AF488 or -AF594 (1:500, cat. 016-540-084; 016-580-084, Jackson Immuno-Research). Alternatively, sections were directly incubated for 2 h at room temperature with the secondary antibodies donkey anti-rabbit AF488 or AF594 (1:500, cat. A-21206, A-21207 Thermo Fisher Scientific). All antibodies were diluted in blocking solution. After staining, tissue was mounted in Fluorescent Mounting Medium (Dako) and pictures taken at Axiovert25 microscope with Axiocam MRm and Axiovision Rel 4.8 software (Zeiss) or with confocal laser scanning microscopy at the Leica TCS SP5 Confocal with LAS AF software (Leica Microsystems). Stereological analysis of vessel density was performed in green fluorescent protein (GFP)-positive tumor area of the CD31- or VWF-positive red fluorescent vessels on every 9th section using the space ball method of the StereoInvestigator Software 10.21.1 (MicroBrightField Bioscience, Williston, VT, USA) connected to an Olympus-BX53-microscope (Olympus Europe, Hamburg, Germany, USA) and a motorized object table MicroBrightField Bioscience.

## Supplementary Materials

Supplementary materials can be found at https://www.mdpi.com/1422-0067/21/11/4179/s1. Figure S1. Modulation of APLN-signaling changes vessel density but not pericyte coverage and endothelial proliferation. (A) U87MG was implanted into immunodeficient mice and grown to big xenografts within 28 dpi. Double-fluorescent immunostaining for CD31+ endothelial cells and Desmin+ pericytes was performed. Double-fluorescent staining confirms decreased vessel density upon reduction of APLN expression in tumour cells (U87^AKD^APLN^WT^), the microenvironment (U87^NSC^APLN^KO^) or both (U87^AKD^APLN^KO^) compared to control (U87^NSC^APLN^WT^). The number of pericyte-covered vessel objects was assessed by the “Count and measure” function in the Cellsense software (Olympus Life Science) on 20× micrographs of different tumour areas of three different tumour sections per animal (six animals per group) and no significant difference of the experimental group compared to the control group was observed (graph not shown). (B) U87MG were grown for 28 dpi and mice intravenously injected with the proliferation marker BrdU three hours before sacrifice. Tumours were stained by double-immuofluorescence for BrdU and Isolectin B4 (IB4) and confocal images were taken. In all four groups BrdU+ endothelial cells (IB4+) could be observed. (C) U87^NSC^APLN^WT^ GBM cells were treated with aCSF or the antagonistic peptide apelin-F13A by intracerebral infusion for 14 days and tumours assessed 28 dpi for vessel density by stereomorphometry (*n* = 6.5). Vessel length density significantly decreased compared to controls when apelin-F13A was used. Data are reported as mean +/-SEM; statistical significance (students t-test) is indicated ** *p* < 0.01. Figure S2. Decrease in *APLN*-expression correlates with increased GBM cell invasion. (A) U87MG cells generally form a very compact tumour 28 dpi (see U87^NSC^APLN^WT^ control in the left panel) but single invading cells (arrows) are especially detectable in U87^AKD^APLN^KO^ xenografts (right panel) by the GFP reporter. By H&E staining a gradual loosening of the tumor border (arrowhead) from left to right panel can be observed (arrows). (B) To assess the extent of GBM cell-invasion, we analysed every 8th tumour section per mouse assigning it an invasive score from 0 to 3 (where 0 is no histological sign of cell invasion from the tumour mass, 1 describes a larger, connected group of invading GBM cells, 2 indicates smaller scattered groups of invading GBM cells and 3 highlights single scattered highly invasive GBM cells). While the U87^NSC^APLN^WT^ control tumours grow fully compact, the invasive score gradually increases when *APLN* expression is knocked down in the tumor cells (U87^AKD^APLN^WT^), the microenvironment (U87^NSC^APLN^KO^) or both (U87^AKD^APLN^KO^). In particular the U87^NSC^APLN^KO^ GBMs exhibit a strong and inter-individually heavily heterogeneous invasive behavior. Here, one set of tumours had moderate levels of invasion (invasive scores below 1, which are comparable to the invasive pattern in the U87^AKD^APLN^WT^ or U87^NSC^APLN^KO^ group), while other gliomas generated by the U87^NSC^APLN^KO^ cells were strongly invasive (invasive scores above 2). (C) Double immunofluorescent staining for GFP-positive tumor (green) and CD31-positive endothelial cells (red) of U87 tumours 28 dpi were performed for U87^NSC^APLN^WT^ or U87^AKD^APLN^KO^ xenografts and merged confocal micrographs (left panels) or a Volocity representation of the confocal z-stacks (right panels) are shown. Note that In the *APLN*-deficient U87^AKD^APLN^KO^ xenografts the GBM cells largely associate with the vasculature which may support directed invasion towards the tumour-free brain. (D) Infusion of Apelin-13 peptide intracerebrally decreased T2-weighted MRI tumour volume of U87^AKD^APLN^KO^ xenografts (*n* = 5) compared to control aCSF treated mice (*n* = 6). (E) Orthotopic GL261 tumor cell implants in APLN^WT^ or APLN^KO^ mice 21 days post implantation show a reduced tumour volume assessed by the Cavalieri Estimator method (StereoInvestigator) and increased tumor cell invasiveness (arrowheads) as quantified on H&E sections (*n* = 9.6 mice). Data are reported as mean +/-SEM; statistical significance (students t-test) is indicated *** *p* < 0.001. (F) Gene expression screen for change in 84 angiogenesis and invasion related genes. RNA was isolated from microdissected orthotpic xenografts of all four U87 GBM groups (*n* = 4 per group) 28 dpi with different APLN expression levels and a qPCR screen using an “RT^2^ Profile PCR Array Mouse Angiogenesis” (Qiagen PAMM 024Z) was performed. Marker genes involved in angiogenesis were downregulated when APLN expression was decreaed in the tumour cells or the microenvironment. Instead the marker for cell invasion MMP2 was found to be upregulated, while its inhibitor TIMP1 was downregulated. Data are reported as mean +/-SEM; statistical significance (one-way ANOVA plus Bonferroni’s post hoc test) is indicated * *p* < 0.05, ** *p* < 0.01.

## Abbreviations

ABP	Average branch points
aCSF	Artificial cerebrospinal fluid
AKD	Apelin knock-down
APLN^KO^	Apelin knock-out
APLN^WT^	Apelin wildtype
APLN	Apelin
APLNR	Apelin Receptor
dpi	days post implantation
GBM	Glioblastoma
GFP	Green fluorescent protein
NSC	Non-silencing control
VEGFA	Vascular endothelial growth factor-A
VLD	Vessel length density
VWF	von Willebrand factor
WT	Wild-type
