# Supplementary material for: Apelin Controls Angiogenesis-Dependent Glioblastoma Growth
Source: Int J Mol Sci. 2020 Jun 11;21(11):4179. doi: 10.3390/ijms21114179 (PMC7312290; doi:10.3390/ijms21114179)
Supplement: Supplementary file 1 [file ijms-21-04179-s001.pdf]

International Journal of
Molecular Sciences

1. Supplementary Materials:

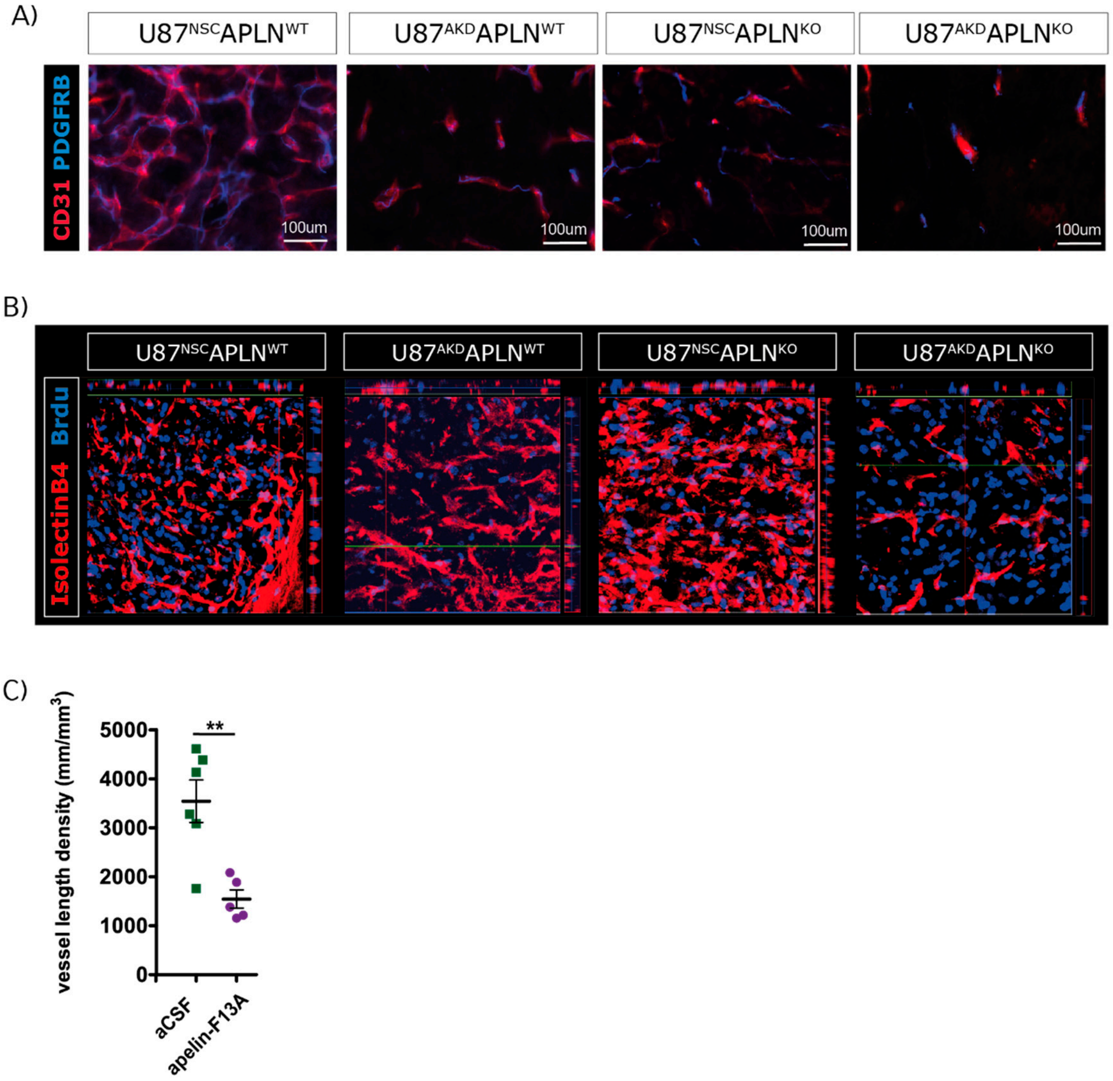

Figure 1. Modulation of APLN-signaling changes vessel density but not pericyte coverage and endothelial proliferation. (**A**) U87MG was implanted into immunodeficient mice and grown to big xenografts within 28 dpi. Double-fluorescent immunostaining for CD31+ endothelial cells and Desmin+ pericytes was performed. Double-fluorescent staining confirms decreased vessel density upon reduction of APLN expression in tumour cells (U87^{AKD}APLN^{WT}), the microenvironment (U87^{NSC}APLN^{KO}) or both (U87^{AKD}APLN^{KO}) compared to control (U87^{NSC}APLN^{WT}). The number of pericyte-covered vessel objects was assessed by the "Count and measure" function in the Cellsense software (Olympus Life Science) on 20× micrographs of different tumour areas of three different tumour sections per animal (six animals per group) and no significant difference of the experimental group compared to the control group was observed (graph not shown). (**B**) U87MG were grown for 28 dpi and mice intravenously injected with the proliferation marker BrdU three hours before sacrifice. Tumours were stained by double-immuofluorescence for BrdU and Isolectin B4 (IB4) and confocal images were taken. In all four groups BrdU+ endothelial cells (IB4+) could be observed. (**C**) U87^{NSC}APLN^{WT} GBM cells were treated with aCSF or the antagonistic peptide apelin-F13A by intracerebral infusion for 14 days and tumours assessed 28 dpi for vessel density by stereomorphometry (n = 6,5). Vessel length density significantly decreased compared to controls when apelin-F13A was used. Data are reported as mean +/-SEM; statistical significance (students t-test) is indicated $^{**}p < 0.01$.

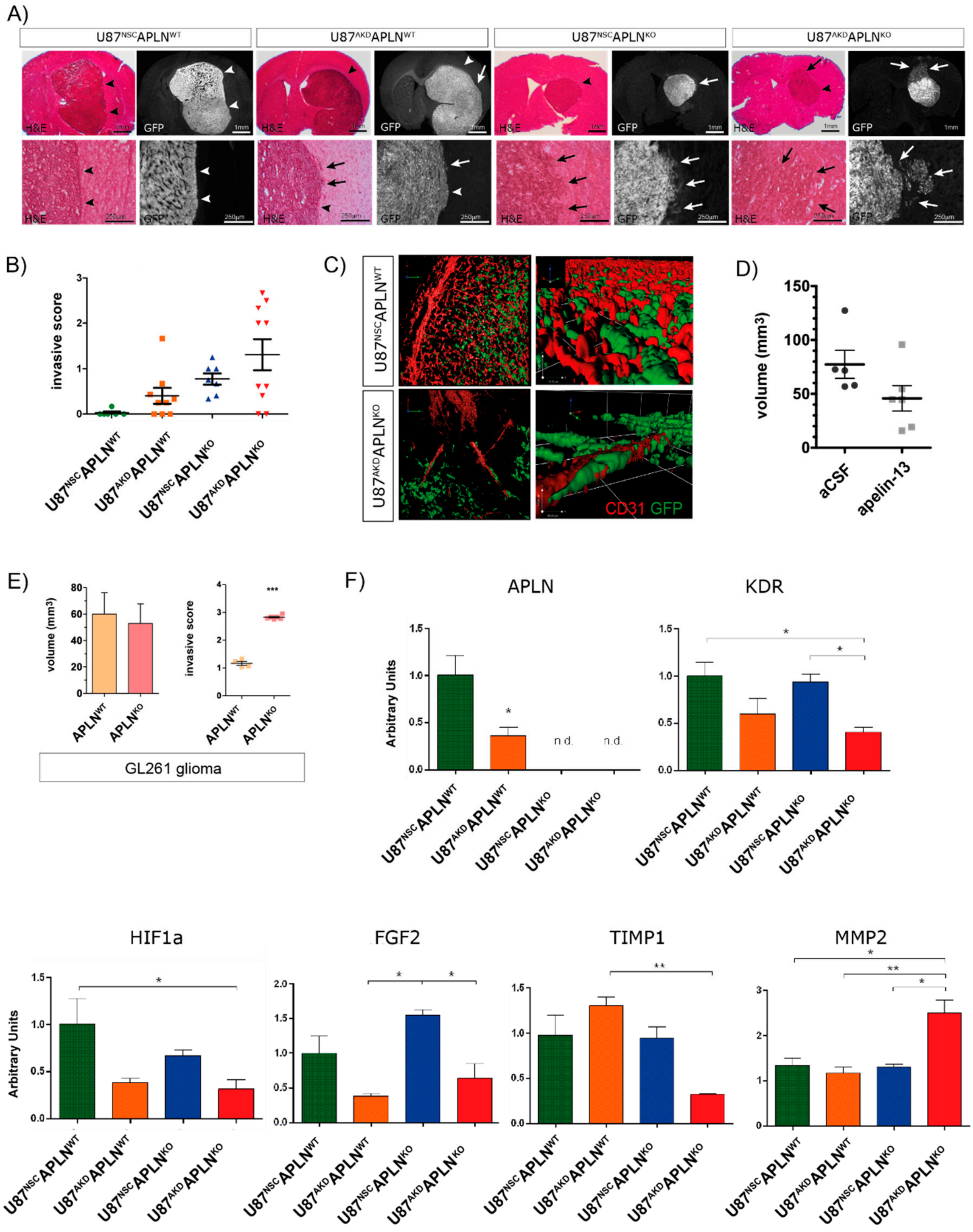

Figure 2. Decrease in *APLN*-expression correlates with increased GBM cell invasion. (**A**) U87MG cells generally form a very compact tumour 28dpi (see U87^{NSC}APLN^{WT} control in the left panel) but single invading cells (arrows) are especially detectable in U87^{AKD}APLN^{KO} xenografts (right panel) by the GFP reporter. By H&E staining a gradual loosening of the tumor border (arrowhead) from left to right panel can be observed (arrows). (**B**) To assess the extent of GBM cell-invasion, we analysed every 8th tumour section per mouse assigning it an invasive score from 0 to 3 (where 0 is no histological sign of cell invasion from the tumour mass, 1 describes a larger, connected group of invading GBM cells, 2 indicates smaller scattered groups of invading GBM cells and 3 highlights single scattered highly invasive GBM cells). While the U87^{NSC}APLN^{WT} control tumours grow fully compact, the invasive score gradually increases when *APLN* expression is knocked down in the tumor cells (U87^{AKD}APLN^{WT}), the microenvironment (U87^{NSC}APLN^{KO}) or both (U87^{AKD}APLN^{KO}). In particular the

U87^{NSC}APLN^{KO} GBMs exhibit a strong and inter-individually heavily heterogeneous invasive behavior. Here, one set of tumours had moderate levels of invasion (invasive scores below 1, which are comparable to the invasive pattern in the U87^{AKD}APLN^{WT} or U87^{NSC}APLN^{KO} group), while other gliomas generated by the U87^{NSC}APLN^{KO} cells were strongly invasive (invasive scores above 2). (**C**) Double immunofluorescent staining for GFP-positive tumor (green) and CD31-positive endothelial cells (red) of U87 tumours 28dpi were performed for U87^{NSC}APLN^{WT} or U87^{AKD}APLN^{KO} xenografts and merged confocal micrographs (left panels) or a Volocity representation of the confocal z-stacks (right panels) are shown. Note that In the *APLN*-deficient U87^{AKD}APLN^{KO} xenografts the GBM cells largely associate with the vasculature which may support directed invasion towards the tumour-free brain. (**D**) Infusion of Apelin-13 peptide intracerebrally decreased T2-weighted MRI tumour volume of U87^{AKD}APLN^{KO} xenografts (n = 5) compared to control aCSF treated mice (n = 6). (**E**) Orthotopic GL261 tumor cell implants in APLN^{WT} or APLN^{KO} mice 21 days post implantation show a reduced tumour volume assessed by the Cavalieri Estimator method (StereoInvestigator) and increased tumor cell invasiveness (arrowheads) as quantified on H&E sections (n = 9, 6 mice). Data are reported as mean +/-SEM; statistical significance (students t-test) is indicated ***$p < 0.001$. (**F**) Gene expression screen for change in 84 angiogenesis and invasion related genes. RNA was isolated from microdissected orthotpic xenografts of all four U87 GBM groups (n = 4 per group) 28 dpi with different APLN expression levels and a qPCR screen using an "RT^2 Profile PCR Array Mouse Angiogenesis" (Qiagen PAMM 024Z) was performed. Marker genes involved in angiogenesis were downregulated when APLN expression was decreaed in the tumour cells or the microenvironment. Instead the marker for cell invasion MMP2 was found to be upregulated, while its inhibitor TIMP1 was downregulated. Data are reported as mean +/-SEM; statistical significance (one-way ANOVA plus Bonferroni´s post hoc test) is indicated *$p < 0.05$, **$p < 0.01$.

© 2020 by the authors. Submitted for possible open access publication under the terms and conditions of the Creative Commons Attribution (CC BY) license (http://creativecommons.org/licenses/by/4.0/).
